# Evaluation of THz wave transmission performance in TOPAS-based heptagonal photonic crystal fiber (He-PCF)

**DOI:** 10.1016/j.heliyon.2024.e25622

**Published:** 2024-02-05

**Authors:** Md Selim Hossain, Rakib Hossen, Md Al-Amin, Sabbir Ahmed, Shuvo Sen

**Affiliations:** a Department of Electronics and Communication Engineering (ECE), Hajee Mohammad Danesh Science and Technology University (HSTU), Dinajpur, 5200, Bangladesh; b Department of Cyber Security, Bangabandhu Sheikh Mujibur Rahman Digital University, Bangladesh Kaliakoir, Gazipur, 1750, Bangladesh; c Department of Electrical and Electronic Engineering (EEE), Daffodil International University (DIU), Dhaka, Bangladesh; d Department of Educational Technology, Bangabandhu Sheikh Mujibur Rahman Digital University, Bangladesh, Kaliakoir, Gazipur, 1750, Bangladesh; e Department of Information and Communication Technology (ICT), Mawlana Bhashani Science and Technology University (MBSTU), Santosh, Tangail, 1902, Bangladesh

**Keywords:** Low EML, Confinement loss, Optical fiber, Single mode H-PCF, THz regime

## Abstract

PCF denotes photonic crystal fiber which is utilized for terahertz (THz) waveguides and cladding in the shape of a hexagon with two elliptical air apertures (AHs), which are discussed. Such differentiation is made: When the frequency is 1 THz, effective material loss (EML) to a minimum of 0.028 cm^−1^ has been achieved. Making use of the heptagonal photonic crystal fiber (He-PCF) architecture, every simulation result utilizing COMSOL Multiphysics software implements the perfectly match layer (PML) and finite element method (FEM) boundary conditions. The He-PCF fiber demonstrates an effective mode loss (EML) of 0.028 cm^−1^ that is negligible, a substantial effective area (EA) measuring 7.31 × 10^−8^ m^2^ and an 80 % power concentration encompassing the central area at 1 THz frequency. Furthermore, regarding crucial optical guiding aspects like confinement loss, dispersion, and modality, a small study with respect to power fraction along with effective mode area (EMA) has again been conducted. Here, He-PCF THz waveguide is anticipated to provide a notable improvement in the current design for the communication field. Moreover, our suggested the PCF demonstrates perception by a solitary mode, as indicated through the utilization of the V-parameter, across a range in frequency spanning among 0.80 and 3 THz. Thus, it is anticipated that the layout of He-PCF fibers will facilitate efficient transmission of terahertz (THz) signals in a variety of communication applications.

## 1 Introduction

Terahertz (THz) radioactivity is not only applicable in optical fiber skill but also in other up-to-date technology [1,2] such as sensing, spectroscopy, drug testing for pharmaceuticals, biological sensing, telecommunications, non-destructive weaponry, DNA hybridization [[3], [4], [5]], etc. THz typeface where there are readily available THz detectors as well; however, the competition among THz waveguides persists because there is currently no established low-loss communication standard that adequately meets the requirements [6]. The design of THz waveguides and their high EML of host material are the biggest challenges. Accurate measurements of Free-electron lasers, Gunn diodes, FIR gas lasers, QCLs, and so forth are crucial. Furthermore, the essential T-ray detectors include Bolometers, Field-effect transistor detectors, hot electron mixers, pair-breaking detectors, Schottky barrier diodes, etc. There are plans to extend the capabilities of this framework, which have grown because of the extra bandwidth available for the current communication infrastructure. This is largely attributed to the dependence on a free space medium, yet most THz waveguides are aware of path loss and absorption loss; additional data is gathered to address gaps that are apparent in our data [7]. We constructed a PCF shape and investigated this PCF in the sense of cholesterol while considering the practical compensations of apparatus based on PCF and the importance of accurate cholesterol identification. The core section contains an expired liquid sample. The primary advantage of photonic crystal fiber (PCF) techniques lies in their ability to enhance sensing capabilities by manipulating geometric parameters such as hole shapes, sizes, and placements [8,9].

In the past few years, there have been reports on a wide variety of dielectric and metallic waveguides that have been specifically designed for operation in the terahertz (THz) spectrum, including Bragg fibers [6], Dielectric glass tubes with a metal coating [10], plastic ribbon waveguides, exposed single metallic filaments, parallel-plate waveguides, and subwavelength porous fibers [[11], [12]]. Due to their adaptable structural design and advantageous optical guiding characteristics, recent interest has been focused on porous core PCF stands for photonic crystal fibers because of their minimal bending loss, elevated birefringence, high nonlinearity, along with lower effective material loss, among other advantages. Two fundamental characteristics of PCF that facilitate light transmission are the photonic bandgap (PGB) and TIR. Optimization is achievable for the comprehensive internal reflection when light passing through solid-core PCF is limited to a higher refractive index region. Numerous types of polymer compounds possess work experience have been employed to be material substrates micro structured photonic crystal fibers (PCF) at their nucleus for regulating the guide for optics characteristics, including Polymethyl methacrylate (PMMA), TOPAS, Tellurite, ZEONEX, Graphene, Teflon, and so forth [[13], [14], [15]].

Numerous standard publications about PCFs have already been investigated to achieve greater EA for efficient transmission and many communication applications over the entire EM spectrum. Using photonic crystal fibers with porous cores and spiral shapes were foreseen by Islam et al. [16]. (PCF). At 1 THz frequency, their suggested model produced the respective EML and EA values are 0.1 cm^−1^ and 1.82 × 10^−7^ m^2^. However, their suggested model displayed more substantial EML. Hasan et al. [17] investigated hexagonal PCFs in 2016 with a gain of 0.089 cm^−1^ in EML at a 1-THz frequency. A dispersion of 0.25 ps/THz/cm accompanied by an EML of 0.053 cm^−1^ was produced by Saiful et al. [18] using their suggested rotating porous hexagonal core and circular form cladding. Regarding 2018, Rana et al. [19] suggested combining an internal hexagonal aperture of a Kagome lattice PCF. At 1.3 THz frequency, their suggested model exhibits with an EML of 0.029 cm^−1^ and a 33 % core power proportion. In the identical year, Sultana et al. [20] created a hexagonal cladding containing a PCF with an elliptical interior to accomplish a very high birefringence of 0.086 where EML is 0.05 cm^−1^. A PCF covered in kagome lattice with an oval core where EML is 0.056 cm^−1^ and Saiful et al. [21] predicted a smaller diffusion of 0.27 ± 0.18 ps/THz/cm at 1 THz operational frequency in 2019. Comparing previously published articles [[16], [17], [18], [19], [20], [21]] reveals that designing and altering the structures of PCF to attain optimal optical attributes within the region of terahertz (THz) frequency has considerable potential.

As of the research, we introduce a heptagonal-shaped PCF based on TOPAS that operates in the THz regime and features an elliptical core. The proposed model exhibits an expansive effective region of 7.31 × 10^−8^ m^2^ at an optical frequency of 1 THz and an effective mode loss (EML) of a negligible 0.0128 cm^−1^ is observed, accompanied by a core that contains 80 % of the total power applied. Furthermore, with respect to the preceding assessment [[22], [23], [24], [25], [26], [27], [28], [29]]**,** with absolute certainty, we are able to optimize the geometric design of the proposed structure and support tangible implementations, including those that involve transmission and communication. This PCF is suitable for numerous terahertz-based communication applications.

## 2 Design methodology

We employ a pitch and diameter from our design concept to define P_1_ and m_1_ as depicted in Fig. 1, the H-PCF is examined in cross-sectional views. TOPAS backdrop material aids in reducing a variety of losses, and the limitations m_1_/P_1_ are known as the air filling ratio, which works to prevent collapse between two AHs. The two elliptical air hole's itch and diameters are referred to as P_c_, m_a_, and m_b_ restrictions, respectively. Here, using the COMSOL Multiphysics program, we determine the numerical characteristics of the fiber in the THz region, encompassing confinement loss, V-parameter, effective area, EML, and scattering loss, as well as the power percentage of fiber. Due to its advanced refractive index, which results in increased mode area confinement within the core region, TOPAS is selected as the bulk substance for this investigation. Besides, it contains minimal bulk substance absorption up to 0.2 cm^−1^. In this context, the specified ideal constraints include cladding diameters (m_1_, m_2_, m_3_, m_4_, m_5_) set at 340 μm, Cladding surfaces (P_1_, P_2_, P_3_, P_4_, P_5_) at 450 μm, and the dimensions of the cores (m_a_, m_b_) at 75 μm and 200 μm, respectively. Additionally, the core pitch (P_c_) is defined as 100 μm. In this scenario, the value of the precisely matched layer's (PML) thickness, which defines computation of the boundary condition as 10 % concerning the greatest fiber diameter. Consequently, PML_1_ is established at 2200 μm, and PML_2_ is set at 2420 μm.

**Fig. 1a:**
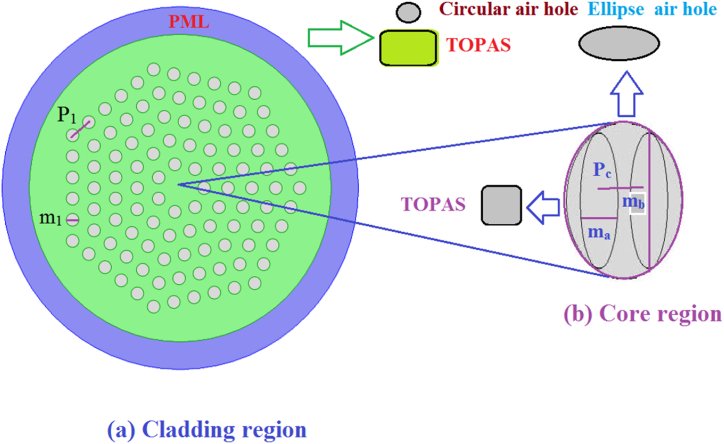
[1 (a–c)]: The H-PCF is illustrated visually in conjunction in relation to the (a) Cladding region, (b) Core region and (c) Distribution of the Mode field.

A laser beam passes through the analytic PCF core in Fig. 1(d), during which the computer acquires data as measured by an optical spectrum analyzer (OSA) in consideration of the intent of analyzing the optical attributes including, but not limited to, loss of efficient materials, perimeter, and effective area. The computer will then perform a numerical and graphical representation of the data.

**Fig. 1b:**
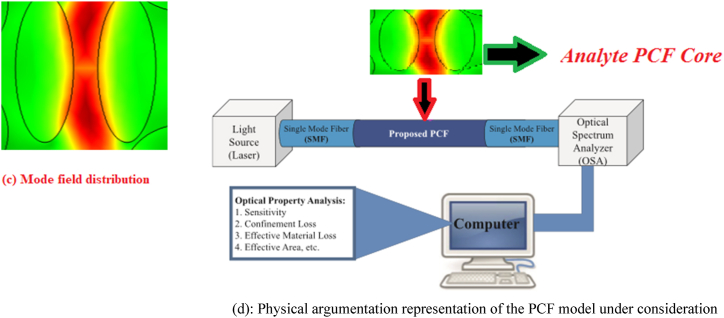
(d):Physical argumentation representation of the PCF model under consideration [12].

## 3 Numerical analysis

To lower the EML, In the production of H-PCF fiber, the substance TOPAS was utilized. Here, EML $\alpha_{eff}$ is premeditated through [30].

(1) $$\alpha_{effective}=\sqrt{\frac{{Ɛ}_{0}}{\mu_{0}}}\left(\frac{\int_{\text{mat}\;}n_{mat\;{\left|E\right|}^{2\;\alpha_{\text{mat}}}}\;dA}{\left|\int_{all}S_{z}dA\right|}\right)\left({\text{cm}}^{-1}\right)$$

Here, n_mat_ represents the material's RI and $\alpha_{mat}$ represents the cumulative waste of materials from absorption. Phosphorus of the atmosphere equates to ${\;\mu }_{0}$ and relative permittivity is equal to ${Ɛ}_{0}$. S_z_ = $\frac{1}{2}$ (E $\times H^{*}$). The vector of pointer, represented by the symbol z, consists of its components S_z_, E, and H*. The symbols S_z_, E, and H* represent the magnetic field's complex dyad component and the electric field component, in that order.

By utilizing the subsequent equation [31], one can determine the SL of H-PCF fiber.

(2) $$\alpha_{R}=C_{R}\times {\left(\frac{f}{c}\right)}^{4}\left(\frac{dB}{km}\right)$$

C_R_ represents the concentration of the scattering coefficient.

PCF fiber with minimal confinement loss is extensively used in a variety of communication applications. In this case, equation utilization yields the confinement loss L_c_ [32]:

(3) $${\;L}_{c}{=8.686\times K}_{0}\;Im\;\left[n_{eff}\right]\;\left(dB/m\right)$$

In the given system, K_0_ = (f/c) denotes the number of unconstrained waves, c signifies velocity of the particle, and the frequency denoted by f. Im [n_eff_] denotes ERI's fictitious component.

The principal constituent of H-PCF fiber is EMA denotes the effective mode area. EMA calculation is performed here using [33].

(4) $$A_{\text{effective}}=\frac{{[\int I\left(rt\right)rtdrt]}^{2}}{{[\int I^{2}\left(rt\right)drt]}^{2}}$$

Here, I (rr) = |Ert|^2^ represents how strong the electromagnetic field is across a section and A_effective_ represents the EMA.

The fraction of power (PF) that is derived from the entire power that traverses the fiber H-PCF. Thus, intends that PF*η* [33]:

(5) $$\eta =\frac{\int_{i}S_{z}dA}{\int_{all}S_{z}dA}$$

The V-parameter delineates the manner in which the H-PCF structure propagates. This means that the following equation limits the V-parameter [34]:

(6) $$V=\frac{2\pi rf}{c}\sqrt{n_{co}^{2}-n_{cl}^{2}}\leq 2.045$$

The respective n_co_ and n_cl_ of the core and cladding area, are indicated by EMI sign when r represents the distance around the nucleus.

## 4 Result analysis and discussions

**Fig. 2:**
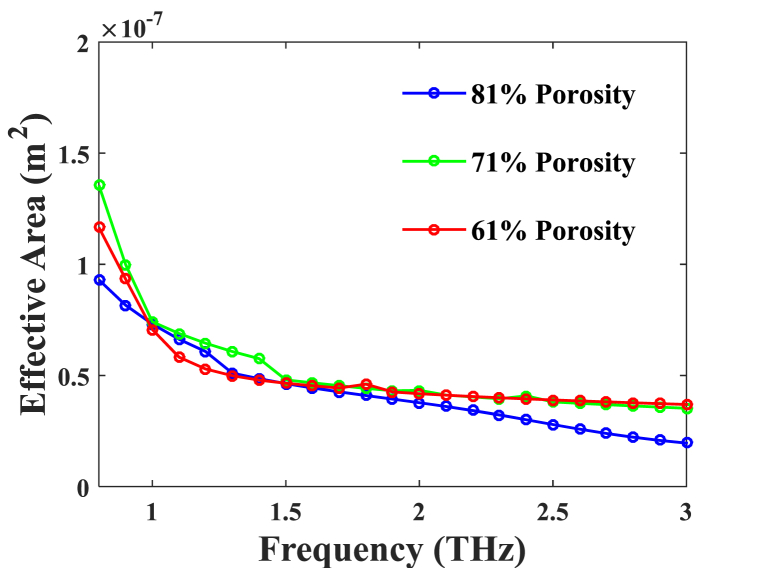
EA based on several frequencies, including 81 %, 71 %, and 61 % porosities.

**Fig. 3:**
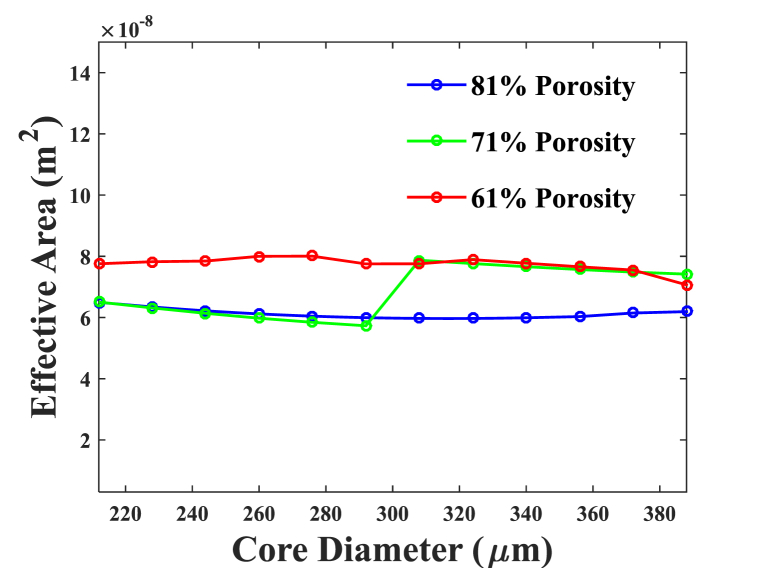
Area effective in accordance with the numerous core diameters, with porosities of 81 %, 71 %, and 61 %, respectively.

**Fig. 4:**
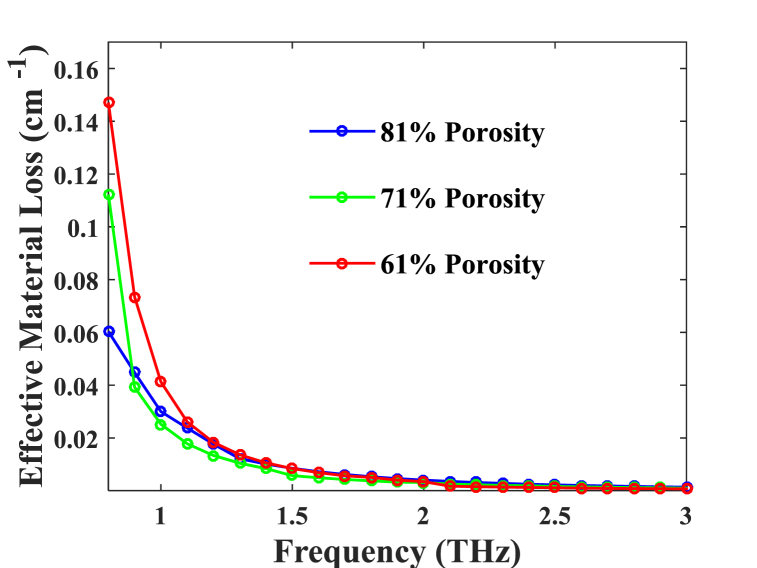
According to frequency, EML has porosities of 81 %, 71 %, and 61 %.

**Fig. 5:**
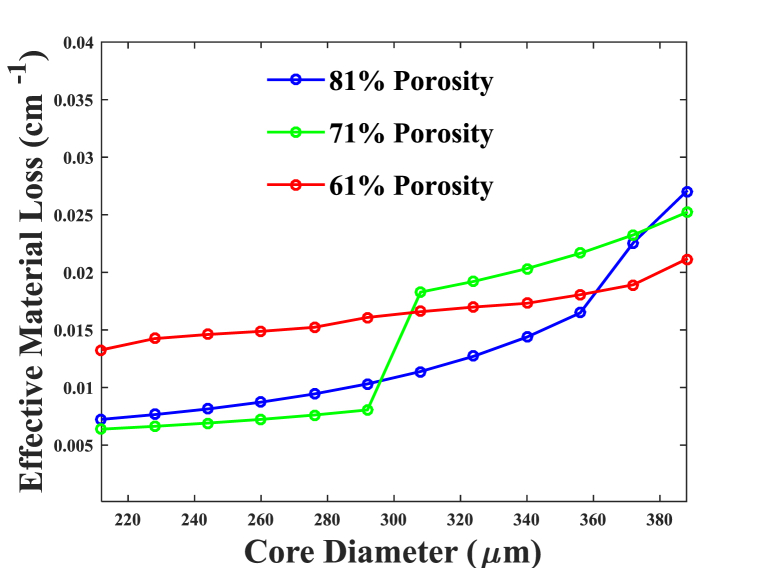
EML based on core diameters with porosities of 81 %, 71 %, and 61 %.

**Fig. 6:**
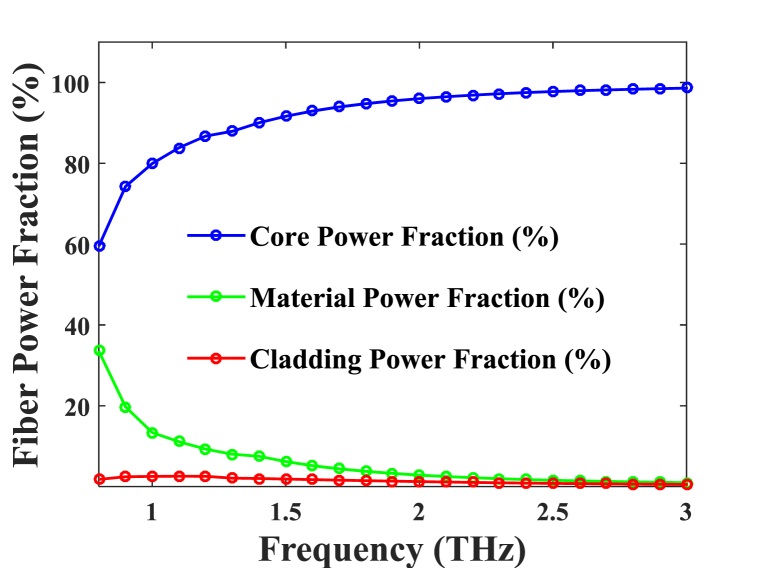
Power fraction for optimum constraints according to various frequencies.

**Fig. 7:**
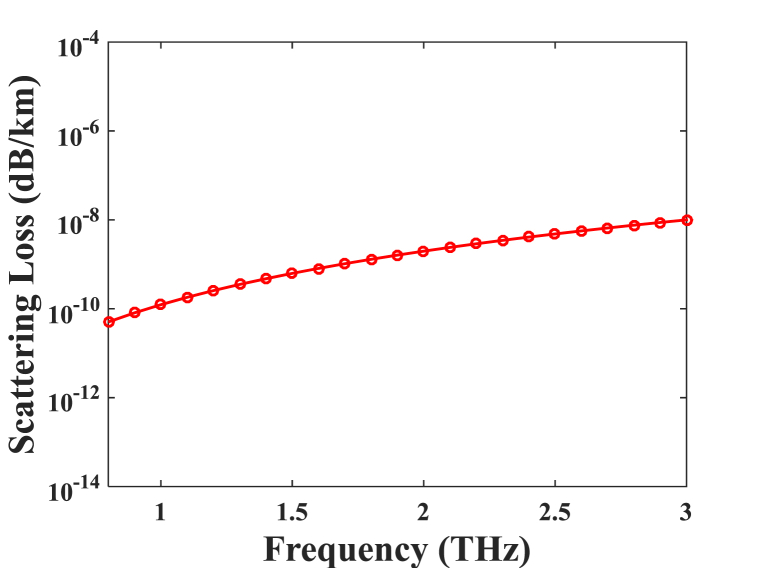
In accordance with the different frequencies, scattering loss for optimal design parameters.

**Fig. 8:**
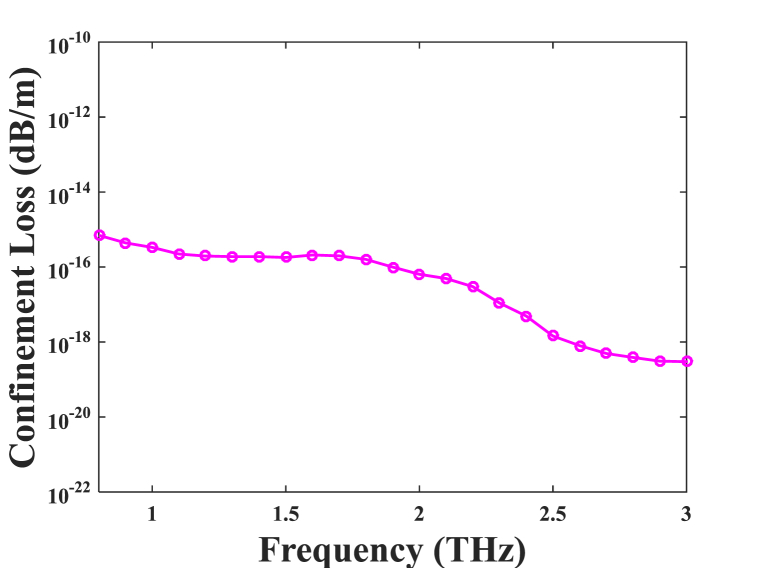
The consequence of confinement for optimal design parameters in consideration of the different frequencies.

**Fig. 9:**
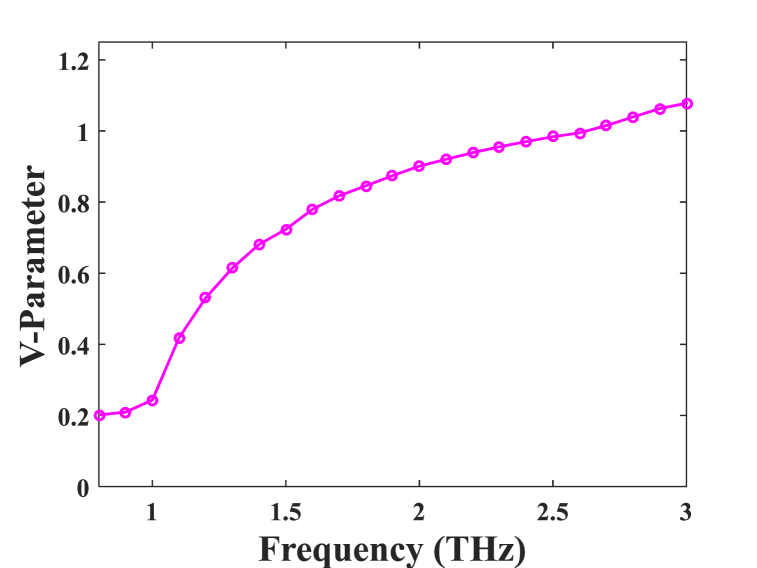
V-parameter for the best design parameters according to the various frequencies.

The principal simulator operates between 0.8 and 3 THz shares the COMSOL Multiphysics software is planned out to perform the calculation of every optical property as well as the visual outcomes spanning Fig. 2, Fig. 3, Fig. 4, Fig. 5, Fig. 6, Fig. 7, Fig. 8, Fig. 9 with regard to the proposed H-PCF. At the transition of frequency from 1 to 3 THz, an illustration of the EA of the intended PCF is provided in Fig. 2 for 61 %, 71 %, and 81 % porosities. It is practical to gradually reduce the region of the effective mode as depicted in Fig. 2. The computed effective areas are as follows: 7.31 × 10^−8^ m^2^ for 81 % porosity, 7.41 × 10^−8^ m^2^ for 71 % porosity, and 7.06 × 10^−8^ m^2^ for 61 % porosity.

Fig. 3 explains the EA for 61 %, 71 %, and 81 % porosities at 1 THz according to diameter of core (D_core_). In order to determine effective area, 6.22 × 10^−8^ m^2^, 6.13 × 10^−8^ m^2^, and 7.84 × 10^−8^ m^2^ for 81 %, 71 %, and 61 % porosities, respectively, for an effective frequency of 1 THz for an ideal core diameter D_core_ = 372 m.

A graph representing effective material loss (EML), frequency-based for various porosities is displayed in Fig. 4. The chart shows that when frequency augments within the range between 0.08 and 3 THz within the electromagnetic range, the proposed structure exhibits a reduction in EML. Consequently, as the core porosity of PCF increases, the quantity of material with which the electromagnetic wave interacts diminishes, leading to a decline in the EML of the proposed fibers. Under optimal conditions throughout the 1 THz range, EMLs, or effective mode losses, are determined to be 0.028 cm^−1^, 0.025 cm^−1^, and 0.041 cm^−1^ corresponding to porosities of 71 %, 61 %, and 81 %.

EML at 1 THz frequency due to differences in the suggested model's core diameter (D_core_) with 61 %, 71 %, and 81 % porosities is depicted in Fig. 5. The EML steadily decreases as core diameter grows when the design parameter is optimal. Considering 81 % core porosity as detailed in our PCFs, approximately 0.028 cm^−1^ is the EML with D_core_ = 372 m, which is the ideal value and does not require any complicated fabrication techniques. The suggested model displays the varied values of EML for various porosities at a constant value of D_core_.

The frequency-dependent the allocation of energy throughout materials, the core, and the cladding is shown via Fig. 6 for a given D_core_ = 372 m. The electromagnetic spectrum's frequency of experiment ranges between 0.08 and 3 THz. Particularly upon its revelation, 80 % at One terahertz in terms of optical output produced via the nucleus of fibers, this indicates which constitutes the primary region receives the greatest light-reaction with analytes. AHs in the cladding region also caused waves of light to travel inside providing the greatest core power fraction and generating the core. Comparing the fraction of pragmatic authority to the first piece, it is noticeably higher.

The analysis of scattering loss in relation to wavelength variations for the proposed structure is depicted in Fig. 7. Scattered loss is an essential metric due to its contribution to the overall losses of the fiber. According to Fig. 7, where D_core_ = 372 m, scattering loss increases as frequency increases in the 0.08 to 3 THz range. The proposed PCF's acquired scattering loss, which is extremely small at optical wavelength 1 THz, is 1.26 × 10^−10^ dB/km.

The methodology of CL in accordance with frequency at the optimal parameter for design is shown in Fig. 8. Between 0.08 and 3 THz, Concerning the confinement loss (CL) of the suggested model diminishes as one increases frequency. When traveling via the core, radiation during a period of elevated frequency increases comparative index that exists between the cladding and the core alongside decreases confinement loss. The anticipated elimination of structure confinement under optimal design constraints at one THz is 3.33 × 10^−16^ dB/m.

Fig. 9 illustrates the investigation of V_eff_ as the frequency function corresponding to the ideal enterprise constraint at D_core_ = 372 μm. Therefore, the PCF under consideration operates under a single mode circumstance, thereby serving given a single mode condition. In this context, the specified ideal constraints include cladding diameters (m_1_, m_2_, m_3_, m_4_, m_5_) set at 340 μm, cladding surfaces (P_1_, P_2_, P_3_, P_4_, P_5_) at 450 μm, and the dimensions of the cores (m_a_, m_b_) at 75 μm and 200 μm, respectively. Additionally, the core pitch (P_c_) is defined as 100 μm.

At 1 THz functional frequency, the designed H_e_-PCF outperforms other designed PCFs pertaining to EML. The effective region's properties, constraints, and core power fraction are as specified in the first table.

In Table 1 we can see that the research results are better than the previous research work. Our discovery was enclosure loss: 3.33 × 10^−16^ dB/m and the effective area: 7.31 × 10^−8^ m^2^, The EML: 0.028 cm^−1^, and the power fraction: 80 % within the monitoring range of 1 THz. In conclusion, the process of fabrication is indispensable component of every style. Practically, A multitude of fabricating processes are utilized to create PCF, including sol-gel, stack and draw, and tube stacking drilling [[27], [28], [29]]. Nevertheless, the sol-gel [35] technique facilitates the structure of PCF generation. As a result, we expect that sol-gel methodology will be more appropriate for producing the PCF fiber that we have designed.

**Table 1:** Comparative propagation between previously proposed PCFs and the He-PCF.

Ref.	EML (cm^−1^)	Porosity (%)	Power Fraction	Confinement Loss (dB/m)	Effective Area (A_eff_ (m^2^))
[22]	0.110	–	–	–	0.98 × 10^−07^
[23]	0.100	30	–	1.0 × 10^−01^	2.3 × 10^−07^
[24]	0.1	–	32.5 %	–	–
[25]	0.085	–	37 %	–	–
[26]	0.05	–	67.05 %	–	–
[27]	0.063	–	46 %	–	–
[28]	0.089	60	37 %	1.0 × 10^−02^	9.77 × 10^−08^
[29]	0.076	80	53 %	8.96 × 10^−01^	–
Proposed H_e_-PCF	0.028	81	80 %	3.33 × 10^−16^	7.31 × 10^−08^

## 5 Conclusion

For communication fields, a superb design with seven layers of heptagonal-based CAHs and in the main region, two elliptical AHs are suggested. This design reduces Numerous types of losses, such as, EML, losses due to confinement and dispersal. TOPAS is background substantial to offset various losses compared to the earlier studies. At this location, a favorable achievement of the EML is 0.028 cm^−1^, which is quite low and was realized within the range of frequencies occupied by 1 THz. The COMSOL Multiphysics simulator was utilized to generate all simulation results in accordance with the heptagonal photonic crystal fiber (He-PCF) concept, employing the boundary conditions of the Finite Element Method (FEM) and the Perfectly Matched Layer (PML). The Heptagonal Photonic Crystal Fiber (H_e_-PCF) exhibits a notable Effective Area (EA) of 7.31 × 10^−8^ m^2^ as well as 0.028 cm^−1^ is a comparatively low Effective Mode Loss (EML). Moreover, at a 1-THz frequency, the core region contains 80 % of the power. Furthermore, our proposed Heptagonal Photonic Crystal Fiber (H_e_-PCF) exhibits additional favorable attributes, including standard confinement facilitated by 3.33 × 10^−16^ dB/m of confinement loss and a low Effective Mode Loss (EML) of 0.028 cm^−1^, and a significant concentration of power in the core at 80 %. Hence, this projected H_e_-PCF would be an auspicious applicant, IoT-based wireless communication is currently being studied in the realm of telecommunications.

## Declarations

The authors declare that they have no known competing financial interests or personal relationships that could have appeared to influence the work reported in this paper.

## Ethical approval

No unethical or bad impact on animals has been done on this work. With the consent of all authors, the paper was submitted to this journal.

## Funding information

No funding has been provided to the authors in support of this study.

## Availability of data and materials

On the request of the correspondent author, data may be provided.

## CRediT authorship contribution statement

**Md Selim Hossain:** Writing – review & editing, Writing – original draft, Validation, Formal analysis, Conceptualization. **Rakib Hossen:** Writing – review & editing, Validation, Methodology, Data curation. **Md Al-Amin:** Writing – original draft, Supervision, Resources. **Sabbir Ahmed:** Writing – review & editing, Visualization, Data curation. **Shuvo Sen:** Visualization, Methodology, Formal analysis, Conceptualization.

## Declaration of competing interest

The authors declare the following financial interests/personal relationships which may be considered as potential competing interests:Shuvo Sen reports was provided by Mawlana Bhashani Science and Technology University. Shuvo Sen reports a relationship with Mawlana Bhashani Science and Technology University that includes: non-financial support. Shuvo Sen has patent pending to NA. S. Sen If there are other authors, they declare that they have no known competing financial interests or personal relationships that could have appeared to influence the work reported in this paper.
